# A Rural-Urban Comparison of Knowledge, Risk- Factors and Preventive Practices for Colorectal Cancer among Adults in Lagos State

**DOI:** 10.31557/APJCP.2019.20.4.1063

**Published:** 2019

**Authors:** Oluwakemi Odukoya, Modupeola Fayemi

**Affiliations:** *Department of Community Health and Primary Care,College of Medicine,University of Lagos, Idi-Araba, Lagos State, Nigeria.*

**Keywords:** Knowledge, adult, colorectal cancer, risk-factors, prevention and practice

## Abstract

**Objective:**

To assess and compare the knowledge, risk-factors and preventive practices for colorectal cancer among adults in Lagos State.

**Material and methods:**

This was a cross-sectional comparative study conducted among 607 respondents selected from one rural (Ikorodu) and one urban (Surulere) LGA using a multistage sampling technique. Data were collected using a pre-tested questionnaire administered by trained research assistants between April and September 2017.Data was analyzed using Epi-info statistical software version 3.5.1. Univariate and bivariate analysis was carried out and -p values of ≤0.05 were considered statistically significant.

**Results:**

Respondents’ knowledge of colorectal cancer was generally low, (rural-78.2%, urban- 62.2%, p<0.001). Urban respondents were significantly more knowledgeable than their rural counterparts (rural- 21.8%, urban- 37.8%, p<0.001). The presence of CRC risk-factors were higher among urban respondents (urban-49.3%, rural-42.6%, p= 0.09), however this difference was not statistically significant. Preventive practices were generally poor in both groups, although more (18.1%) urban respondents significantly took preventive actions against CRC compared with rural (6.9%) respondents, (p<0.001).Increasing levels of education were significantly associated with higher knowledge level in both groups (p≤0.05).

**Conclusion:**

The level of knowledge of colorectal cancer was generally poor in both groups but significantly poorer among rural respondents. The presence of known risk- factor was higher among urban respondents while preventive practices were poor in both groups.

## Introduction

Colorectal cancer (CRC) is an important public health issue. It ranks fourth in the most common cause of death due to cancers worldwide (WHO, 2012) and is at present one of the top five cancers prevalent in Nigeria (GLOBOCAN, 2012). CRC, commonly known as colon cancer or bowel cancer, is due to uncontrolled cell growth in the colon or rectum, or in the appendix (Al-Jashamy, 2013).

The development of CRC may be linked to several factors, among which are hereditary and environmental factors (America cancer society, 2014). Although the etiology is not known, CRC is considered a multifactorial disease, an important role being attributed to the impact of environmental factors on a genetically prone land. A hypercaloric diet, high in fat and low in dietary fiber is positively correlated with the CRC occurrence. Obesity, western diet and lack of physical activity are common risk factors for CRC (Chalya et al., 2013) Furthermore, smoking, drinking and non-alcoholic fatty liver disease increases the risk of CRC (Ng and Wong, 2013)

Early CRC often has no symptoms, which is why secondary preventive measures are so important. CRC may be easily prevented through CRC screening which can detect the disease during its early stages when the survival rates are highest (Christou and Thompson, 2012).Unfortunately, CRC screening (CRCS) uptake is lower than that of other screening-amenable cancers worldwide (Joseph, 2012). In Nigeria, despite the availability of resources for diagnosis of CRC; there is lack of consistent organized screening programs on the national level.

This observed increase in the CRC cases reported in the country has been hinged on change in the hitherto rural lifestyle to the more urban type (Irabor, 2012). The geographic patterns of CRC have indicated positive correlations with urbanization, socioeconomic status, and the “western” type of diet (Correa and Haenszel, 1978). In Nigeria, rural and urban lifestyle are characterized by the choices and preferences of individuals in these areas, a study carried out on food consumption patterns reported that certain dishes are perceived as “poor people’s food” or “rich people’s food”(Obayelu et al., 2009). For example, the consumption of food such as gari, smoked fish, cassava flour, hide and skin (locally known as “Ponmo”) etc are considered as food for poor people by some urban dwellers even though they are highly nutritious as argued by some nutritionists (Obayelu et al., 2009). The consumption of such foods has therefore strong resistance by some households in the urban areas because of the social class perceptions attached to them. Urban residents in the study areas purchased 37.9% of the food they consume, while families in rural areas purchased only 26.6% (Obayelu et al., 2009). Furthermore, urban lifestyle tends to be associated with higher educational level, medical insurance, healthy or risky behaviors, and access to care (i.e., proximity to healthcare facilities, a regular source of care) (Onibokun and Faniran, 1995; Nigeria bureau of statistic, 2008). Individuals in rural areas tend to be less educated, have a lower income, lack health insurance, and travel a distance to access health care compared to their urban counterparts. 

CRC was considered to be rare in Africa three to four decades ago, which was credited to the starchy, high-fiber, spicy, peppery foodstuff low in animal protein which many West African nations consume (Berkowitz et al., 2008). However, with increasing change in eating culture and westernization, this happens no longer to be true. Population based studies are lacking in Nigeria however, studies carried out in Nigerian teaching hospitals reported high level of CRC (Sung et al., 2008) also the surgical biopsy registry at Lagos University Teaching Hospital (LUTH) has identified CRC as the third most commonly diagnosed malignancy (Sung et al., 2008; Abdulkareem, 2008).

The Nigeria society is rapidly becoming urban, because of multiple factors like possibilities of improved access to jobs, goods and services, shelter, stability, prosperity, security, social inclusion and more equitable access to the other services (Muggah, 2012; Celik et al., 2009). Evidence of this is the increase shift of population from rural to urban areas and an increase in the number of people living in an urban area at a particular time (Akunnaya and Adedapo, 2014). In 1950, only 10.1% Nigeria population was urban, this rose to 20.0% by 1970, 43.3% in 2000, and it is expected to reach 58.3% by 2020 (Onibokun and Faniran, 1995). Few studies have assessed population level knowledge of CRC and the presence of known CRC risk-factors and preventive practices and there are virtually no studies that have compared these among rural and urban respondents. This study therefore set out to assess and compare the knowledge, risk-factors and preventive practices for colorectal cancer among adults in Lagos State.

## Materials and Methods

*Study Setting, population, design and sample determination*

Lagos is the smallest state in Nigeria with a land mass of 3.557 km^2^. It has 20 local government areas (16 Urban and 4 rural). The study was conducted in one rural (Ikorodu) and one urban (Surulere) LGA in the State.

Ikorodu is a city in north-east Lagos State; it is situated approximately 36 km north of Lagos, Nigeria. At the 2006 census the population was 535,619. The local government has 19 wards. The main occupation of Ikorodu people are Trading (commerce) and farming. The area is characterized by several towns and villages with untarred roads, poor infrastructural development and a high level of traditional practices. The tarred roads are the ones linking Ikorodu to the city. 

Surulere Local Government Area is located on the mainland Lagos State, Nigeria. It has an area of 23 km²with a population of 503,975 at 2006 census. The local government has 9 wards. Surulere is a residential and commercial area, it boosts of national stadium, malls, entertainment outlets, plaza, restaurants, tarred roads and good infrastructural development. It has a highly cultural diverse population with diverse professions as well. 

*Methods*

The cross-sectional comparative study was conducted among adult residents in both LGA’s. Eligible respondents were aged 18 years and above and must have resided in the area for at least six months. The minimum sample size of 304 respondents per local government was determined using the standard formula for determination of the sample size for comparative studies; with A=95% significance level and a power of 80%. A proportion of respondents with Knowledge of CRC of 32% and 46% in rural and urban areas respectively. A 20% non-response rate was added resulting in a final sample size of 304 per group.

*Sampling methods and data collection tools and techniques*

A multistage sampling technique was used to select the respondents who were interviewed by trained research assistants after an informed consent was obtained. In the first stage we obtained a sampling frame of 20 local governments (16 urban and 4 rural) and selected one rural and one urban by simple balloting. The second stage involved the selection of wards. There are nineteen (19) wards in Ikorodu and nine (9) wards in Surulere LGA. The list of the wards in each of the selected local governments was obtained from the Information unit of the local government office. Six (6) wards each were selected from the above selected local government areas using simple random technique. The sample size was evenly distributed across the 12 wards. In the third stage streets were selected from the selected wards in the two LGA. Five streets were selected using simple random technique in each ward from a list of streets obtained from the respective Local government offices. The fourth stage involved the selection of houses; Systematic sampling method was used to select 10 houses each from the five streets for the urban area. Each of the streets has about 60 houses, every 6^th^ house was considered from the beginning of the street i.e N/n= 60/10 = 6. The streets in the rural area have about 40 houses, to get 10 houses; every 4th house was considered starting from the beginning of the street. i.e N/n = 40/10 = 4. In the final stage we selected eligible respondents (Adults living in Ikorodu and Surulere local government area during the data collection period (June-August) and who has resided in the area for at least six months) from the households in the urban and rural LGA using simple balloting.

A pretested interviewer-administered questionnaire developed from a review of relevant literatures and adapted from validated tool such as; Bowel/Colorectal Cancer Awareness Measure (Bowel/Colorectal CAM) questionnaire (Sessa et al., 2008) colorectal cancer risk assessment questionnaire (Aria Jefferson Health, 2016) and a colorectal cancer screening survey questionnaire (Wolf, 2005) was used to collect data. The questionnaire elicited information on respondents following: A) Socio-demographic characteristics i.e age, gender, religion, ethnic, state of origin, marital status, highest educational level, occupation, daily activity, household size and income B) Knowledge of CRC (knowledge of the following; do you know cancer affects the intestine? Possible signs of CRC questions such as; Bleeding from the back passage, Persistent pain in the abdomen, change in bowel habits over a period of 2 weeks, Blood in stool, Pain in back passage, prevention questions such as, CRC can be prevented by; limiting alcohol intake, eating food low in fibre, screening every 1 or 2 years, was elicited C) CRC risk- factors i.e eating of low fibre diets, drinking of beverage containing alcohol per day, current/previous smoking of cigarette/ tobacco, daily activities involve walking/sitting most of the day, exercising for up to 30 minutes for at least 5 days in a week, family history of cancer, BMI category, D) Preventive measures for colorectal cancer i.e previous history of medical check-up, previous history of CRC screening and preventive actions against CRC. Data collection was carried out between March and September 2017.

*Statistical analysis*

*Scoring*

a) Respondents’ knowledge of colorectal cancer was assessed using 23 questions. Each correct response was scored one mark and any wrong or non-response was scored zero mark. The total score obtained from each respondent was converted to percentage and graded as good (≥50%) and poor (<50%). The mean knowledge score (%) for all the respondents was also calculated. 

b) Respondents risk for colorectal cancer were assessed using 15 questions. For questions whose responses were either yes or no, those who had risk with Yes answer were scored 1 while those who had no were scored 0. For questions with 5 options, it was rated on a 4point likertscale. The most risky factor was scored 4, the next was scored 3, in that other and the least risky factor was scored 1. For questions with 4 options, it was rated on 3point likertscale. The most risky factor was scored 3, the next was scored 2, and the least risky factor was scored 1. In terms of body mass index, those who were normal and underweight scored 0, overweight 1 and obesity 2. Categorization was done using the mean risk factor score. Those with score below the mean were categorized as low risk while those with score above the mean were classified as high risk. 

Date analysis: Data processing was done using EPI-INFO epidemiological software package version 3.5.1 and the Microsoft Excel. Frequency distribution tables were constructed. Chi-square and fisher’s exact test were used for comparison of proportions while t-test was used for comparison of differences between means. P value of ≤0.05 was considered statistically significant.

Univariate and bivariate analysis were carried out. P value ≤0.05 was considered statistically significant.

*Ethical Considerations*

Approval was obtained from the Human Research and Ethics Committee (HREC) of our institution. Permission was obtained from the local government chairman and community leaders. 

Respondents were provided with full explanation of the study with the emphasis on their right of not to participate and written informed consent was obtained from each of them by signing on the questionnaire indicating their willingness to participate in the study. No names were printed on the questionnaires and the participants were assured of the confidential nature of the study. 

## Results

All the 304 questionnaires from the urban respondents were adequately filled and analyzed while 303 out of 304 questionnaires were adequately filled and analyzed by rural respondents, giving a response rate of 100% and 99.9% respectively.

Socio - demographic characteristics of the respondents: The mean ages were 35.40±11.5 and 30.85±11.9 in the rural and urban areas respectively. Rural respondents were significantly older than their urban counterparts. Most of the respondents were female (rural-58.4%, urban-51.6% p=0.09), Christian (rural-69.6%, urban- 61.8% p=0.06), Yoruba (rural-74.9%, urban-69.1% p=0.006), employed (rural-80.2%, urban-84.9% p=0.13) and with at least secondary education (rural-48.8%, urban-50% p= <0.001). More of urban respondents were single (59.9%) compared to rural respondents with more married respondents (58.7%), the difference was statistically significant (p<0.001). Urban respondents had significantly larger families size (urban- 4.98 ±2.36, rural- 4.63 ±1.89), (p=0.04) (Table not shown).

**Table 1:** Assessment and Comparison of the Knowledge on Colorectal Cancer among Rural and Urban Respondents

Knowledge item	Rural n=303	Urban n=304	P-value
	Freq %	Freq %	
CRC is hereditary			
Yes	41 (13.5)	108 (35.5)	<0.001
No	262 (86.5)	196 (64.5)	
Limiting alcohol intake prevents CRC			
Yes	124 (40.9)	169 (55.6)	<0.001
No	179 (59.1)	135 (43.0)	
Eating food low in fiber prevents CRC			
Yes	115 (38.0)	140 (46.1)	0.043
No	188 (62.0)	164 (53.9)	
Being physically active prevents CRC			
Yes	117 (38.6)	156 (51.3)	0.002
No	186 (61.4)	148 (48.7)	
CRC can be detected early by screening			
Yes	120 (39.6)	149 (49.0)	0.02
No	183 (60.4)	155 (51.0)	
CRC can be prevented			
Yes	127 (41.9)	191 (62.8)	<0.001
No	176 (58.1)	113 (37.2)	
CRC can be cured if detected early			
Yes	115 (38.0)	150 (49.3)	0.005
No	188 (62.0)	154 (50.7)	
Grade			
Good	66 (21.8)	115 (37.8)	<0.001
Poor	237 (78.2)	189 (62.2)	
Total	303 (100)	304 (100)	

**Table 2:** Assessment and Comparison of Known Colorectal Cancer Risk Factors among the Rural and Urban Respondents

Risk Factor	Rural n=303 Freq (%)	Urban n=304 Freq (%)	P- value
Family history of cancer			
Yes	20 (6.6)	32 (10.5)	0.084
No	283 (93.4)	272 (89.5)	
Frequency of alcohol consumption			
Never	230 (75.9)	193 (63.5)	0.001
Monthly	42 (13.9)	77 (25.3)	
>3 times a week	31 (10.2)	34 (11.2)	
Number of drinks consumed on a typical day			
<4	74 (24.4)	85 (28.0)	<0.001
>5	8 (2.6)	30 (9.9)	
Never	221 (72.9)	189 (62.2)	
Daily activities involve walking about most of the day			
Yes	162 (53.5)	189 (62.2)	0.029
No	141 (46.5)	115 (37.8)	
Exercise for up to 30 minutes for at least 5 days in a week			
Yes	115 (38.0)	139 (45.7)	0.052
No	188 (62.0)	165 (54.3)	
Currently smoke tobacco			
Yes	31 (10.2)	45 (14.8)	0.089
No	272 (89.8)	259(85.2)	
Previous tobacco use			
Yes	35 (11.6)	46 (15.1)	0.195
No	268 (88.4)	258 (84.9)	
Frequency of consumption of diet low in fiber			
< a week	89 (29.4)	68 (22.4)	<0.001
Once in a week	152 (50.2)	63 (20.7)	
2/3 times in a week	35 (11.6)	59 (19.4)	
4/5 times in a week	13 (4.3)	17 (5.6)	
Always	14 (4.7)	97 (31.9)	
Frequency of consumption of diet high in Fat			
< a week	26 (8.6)	39 (12.8)	<0.001
Once in a week	9 (3.0)	49 (16.1)	
2/3 times in a week	48 (15.8)	71 (23.4)	
4/5 times in a week	174 (57.4)	33 (10.9)	
Always	46 (15.2)	112 (36.8)	
BMI body mass index grouping			
Not overweight/obese	231 (72.6)	208 (68.4)	0.031
Overweight/obese	72 (23.8)	96 (31.6)	
Mean (SD)	23.57±4.66	23.60±4.83	
Risk-factor grade			
High risk (>mean risk-factor score)	129 (42.6)	150 (49.3)	0.094
Low risk (<mean risk factor score)	174 (57.4)	154 (50.7)	
Mean (SD)	11.13±3.92	11.97±4.81	0.019
Total	303 (100)	304 (100)	
Previous history of CRC screening			
Yes	2 (0.1)	8 (2.6)	0.059^F^
No	301 (99.3)	284 (48.6)	
Prevent CRC by cutting down on red meat			
Yes	12 (4.00)	30 (9.90)	0.004
No	291 (96.00)	274(90.10)	
Prevent CRC by increasing physical activity			
Yes	16 (5.30)	23 (7.60)	<0.001
No	287 (94.70)	32 (92.40)	
Prevent CRC by maintaining a healthy weight			
Yes	12 (4.00)	25 (8.20)	0.028
No	291 (96.00)	279(91.80)	
Prevent CRC by avoiding excessive alcohol			
Yes	4 ( 1.30)	18 (5.9)	0.004^F^
No	299 (98.70)	286 (94.10)	
Prevent CRC by ensuring regular bowel activity			
Yes	7 (2.30)	17 (5.60)	0.04
No	296 (97.70)	287(94.40)	
Prevent CRC by eating food high in fiber			
Yes	7 (2.30)	24 (7.90)	0.002
No	296 (97.70)	280 (92.10)	
Prevent CRC by avoiding tobacco use			
Yes	5 (1.70)	13 (4.30)	0.057
No	298 (98.30)	291 (95.70)	
Prevent CRC by getting screened			
Yes	5 (1.70)	8 (2.60)	0.404
No	298 (98.30)	296 (97.40)	
Total	303 (100)	304 (100)	

F, Fishers exact

**Table 3:** Bivariate Analysis of Factors Associated with Colorectal Cancer Knowledge of Rural and Urban Respondents

Socio-demographic	Rural n= 303		P-value	Urban n=304		P-value
variable	Good grade n= 66 Freq. (%)	Poor grade n=237 Freq. (%)		Good grade n=115 Freq. (%)	Poor grade n=189 Freq. (%)	
Age (in years)						
18-25	10 (15.15)	51 (21.52)	0.506	43 (37.39)	71 (37.57)	0.15
26-35	23 (34.85)	97 (40.93)		46 (40.00))	64 (33.86)	
36-45	19 (28.79)	51 (21.52)		18 (15.65)	34 (17.99)	
46-55	9 (13.64)	23 (9.70)		3 (2.61)	12 (6.35)	
56-65	4 (6.06)	9 (3.80)		5 (4.35)	3 (1.59)	
66 and above	1 (1.52)	6 (2.31)		0 (0.0)	5 (2.65)	
	t-test=1.818 p-value= 0.070			t-test=0.929 p-value=0.354		
Gender						
Male	33 (50.0)	93 (39.24)	0.117	66 (57.39)	81 (42.86)	0.014
Female	33 (50.0)	144 (60.76)		49 (42.61)	108 (57.14)	
Ethnicity						
Hausa	5 (7.60)	20 (8.40)	0.813^F^	3 (20.00)	12 (80.00)	0.263
Igbo	9 (19.60)	37 (15.60)		20 (17.40)	41 (21.70)	
Yoruba	52 (78.80)	175 (73.80)		83 (72.20)	127 (67.20)	
Others	0 (0.00)	5 (2.10)		9 (50.00)	9 (50.00)	
Marital status						
Single	18 (27.30)	75 (31.60)	0.656	73 (63.50)	109 (57.70)	0.059^F^
Married	42 (63.60)	136 (57.40)		41 (35.70))	68 (36.00)	
Others	6 (9.10)	26 (11.00)		1 (0.90)	12 (6.30)	
Highest educational level						
< Sec. education	17 (25.80)	90 (38.00)	0.01	3 (2.60)	24 (12.70)	<0.001^F^
Sec education	31 (47.00)	117(49.40)		53 (46.10)	99 (65.13)	
Tertiary	18 (27.30)	30 (12.70)		59 (51.30)	66 (52.80)	
Employment status						
Employed	54 (81.80)	189 (79.70)	0.708	94 (81.70)	164 (86.80)	0.235
Unemployed	12 (18.20)	48 (20.30)		21 (18.30)	25 (13.20)	
Monthly Earnings (₦)						
<18,0000	26 (39.4)	109 (4.0)	0.4	52 (45.2)	92 (48.68)	0.193^F^
18,000- 100,000	21 (52.0)	118 (49.8)		59 (51.3)	82 (43.40)	
>₦100,000	5 (4.6)	10 (4.2)		4 (3.5)	15 (7.90)	
Occupation						
Skilled	34 (51.5)	96 (40.5)	0.232	79 (68.7)	119 (63.0)	0.001^F^
Semi-skilled	18 (27.3)	88 (37.1)		32 (27.8)	39 (20.6)	
Unskilled	14 (21.2)	53 (22.4)		4 (3.5)	31 (116.4)	
Total	66 (100)	237 (100)		115 (100)	189 (100)	

F, Fishers exact

Assessment and comparison of the knowledge of colorectal cancer among rural and urban respondents: About one-third of urban respondents (35.5%) knew that CRC is hereditary compared to about one-seventh (13.5%) of the rural respondents, this difference was statistically significant (p<0.001). More than half of rural respondents (58.4%) were not aware that limiting alcohol intake prevents CRC compared to urban respondents (41.1%), this difference was statistically significant (p<0.001). A higher proportion of urban respondents were aware that CRC can be prevented (62.8%),can be detected early by screening and cured if detected early (50%) compared to rural respondents (41.9%, 40%) respectively, this difference was statistically significant (p<0.001). 

Respondents knowledge of colorectal cancer was generally poor (rural-78.2%, urban- 62.2%), however urban respondents tended to be significantly more knowledgeable (p<0.001) (Table 1).

Assessment and comparison of known colorectal cancer risk factors among the rural and urban respondents: Both groups of respondents had a high risk for colorectal cancer with the urban respondents been significantly higher than rural respondents (urban: 11.97±4.8, rural: 11.13±3.9 p=0.019). More of urban respondents (10.53%) had a family history of cancer compared with rural respondents (6.6%), however this difference was not statistically significant (p=0.084).One-fourth (25.3%) of urban respondents consume alcohol monthly compared with rural respondents (13.9%), this difference was statistically significant (p=0.001). A higher proportion of urban respondents (45.7%) exercise for up to 30 minutes for at least 5 days a week compared with less than half of rural respondents (38%), however this difference was not statistically significant (p=0.052). More of urban respondents (14.8%) currently smoke tobacco compared with rural respondents (10.2%), but the difference was not statistically significant (p=0.195). One-third (31.91%) of urban respondents eat a diet low in fiber always compared with <5% of rural respondents, the difference was statistically significantly (p<0.001). One-fourth (24.01%) of urban respondents were overweight compared with one-sixth of rural respondents (16%), this difference was statistically significant (p<0.001) (Table 2).

Assessment and comparison of preventive measures for colorectal cancer among rural and urban respondents: Respondents practice of preventive actions against CRC was generally poor in both groups, only few of the respondents practiced all the preventive measures indicated for CRC in both areas. More of urban respondents (18.1%) took preventive actions against CRC compared with 6.9% of rural respondents, this difference was statistically significant (p<0.001). About 9.9% of urban respondents prevent CRC by cutting down on meat compared to 4% of rural respondents, the difference was statistically significant (p=0.004). More of urban respondents (5.9%) go moderate on alcohol to prevent CRC compared with rural respondents (1.3%), this difference was statistically significant (p=0.004). About (8.2%) of urban respondents prevent CRC by maintaining healthy weight compared with only 4% of rural respondents, this difference was statistically significant (p=0.03) (Table 2).

Bivariate analysis of the factors associated with colorectal cancer knowledge of rural and urban respondents: Demographic factors associated with the knowledge level of rural and urban respondents showed that knowledge of CRC was not significantly associated with age in either of the LGA’s. In the urban area male respondents (57.39%) were significantly more knowledgeable than female (42.61% p=0.014) compared with rural were both female and male were equally knowledgeable (50%). Education was statistically significantly associated with knowledge of CRC for rural residents (p=0.010) and urban residents (p<0.001) respectively, knowledge increased with education for both groups. Occupation was statistically significantly associated with knowledge level of CRC (p=0.001) for urban respondents, however not significantly associated for rural respondents (p=0.232), majority of urban respondents (68.7%) engaged in skilled occupation had good grades compared to rural respondents (51.5%) (Table 3).

## Discussion

This study revealed generally poor levels of knowledge among the urban and rural respondents as there were large knowledge gaps towards many individual items; A greater percentage of the respondents in rural and urban local governments (78%, 62.2%) respectively had poor grade. This is consistent with the report of other studies carried out in Asia, which reported a considerable body of evidence highlighting low levels of knowledge about CRC in many countries. The Asia Pacific Working Group in Colorectal Cancer conducted a multinational survey in various Asia Pacific regions and detected low knowledge scores for symptoms and risk factors, with quite several regions scoring 0 (koo et al., 2012).

Poor knowledge of CRC is common and not restricted only to the developing or under- developed nations (Von et al., 2007). Studies from developed nations have all shown suboptimal knowledge and awareness for CRC, although better than developing nations (koo et al., 2012; Myers, 2007).This supports the overall poor knowledge reported at the rural and urban local government that was studied, even among those with tertiary education. 

Similar to the current findings in rural respondents, a study carried out among rural respondents in Malaysia reported poorer knowledge of signs of CRC among rural respondents, it was reported that only one-third of the respondents answered questions of signs/symptoms correctly (Tin and Jun, 2013). From this study it was observed that knowledge of possible signs of CRC was better among urban respondents compared to rural respondents as it showed that a higher number of rural respondents (74.6%) were not aware that bleeding from back passage is a possible sign of colorectal cancer in comparison to urban respondents (49.1%), which is similar to a study carried out among rural respondents in Malaysia that reported 6.6% of respondents recognized bleeding from back passage as warning sign of CRC (Tin and Jun, 2013). 

Disparities were observed in the current findings among rural and urban respondents with regards to the possible signs of CRC compared with previous studies; about one quarter (21.3%) of rural respondents knew that change in bowel habits over a period of 2 weeks is a possible sign of CRC which contrasted with the report from a similar study in Malaysia among rural respondents, that reported 3.9% recognized change in bowel habits as warning signs of CRC (Tin and Jun, 2013).When compared with one-third (32.6%) of urban respondents in this study which also contradicts with a similar study among urban respondents in Saudi-Arabia that showed changes in bowel habits was correctly identified by 48% of the respondents (Yasmine et al., 2016). Also, one-fifth (22.1%) of rural respondents knew that blood in stool is a possible sign of CRC compared with two-fifth (41.8%) of urban respondents. This contrasts with findings among rural respondents in a previous study (Tin and Jun, 2013) that reported 6% recognized blood in stool as a warning sign of CRC and similar to a previous study among urban respondents that reported 54.2% correctly identified blood in stool as symptoms of CRC (Yasmine et al., 2016), also about half of urban respondents (46.1%) knew that tiredness/anaemia is a possible sign of CRC compared with rural respondents (21.8%). This contrasts with a previous study where unexplainable weakness was correctly identified by (23.5%) of urban respondents as symptoms of CRC (Yasmine et al., 2016). One can suggest that the observed disparity could be as a result of area of residence among other factors, because a larger percentage of respondents in rural area simply have a negative attitude towards cancer (McCaffery, 2003).

The knowledge of prevention and early detection of CRC was significantly higher among urban respondents with more than half of urban respondents been aware that CRC can be prevented and detected early by screening and that CRC can be cured if detected early, this findings goes in line with the study carried out in an urban area in Italy (Sessa et al., 2008) where 60.3% and 78. 5% responded that it is possible to prevent CRC and to treat the cancer in case of early diagnosis while less than 50% in the rural area answered these questions correctly which is similar to the study carried out in Saudi- Arabia (Osama, 2015) which reported that 37% of participants believed that it is possible to prevent CRC. This can have implication on designing and implementing health campaigns to address the point of preventability of the disease and a total of 43.7% of respondents from the current study believed that CRC can be treated if diagnosed early, which is a good sign in helping to motivate people with implementing screening programs.

Mean risk-factor score was significantly different among rural and urban respondents (p=0.019), (rural-11.13±3.92; urban- 11.97±4.81). More of urban respondents (50.7%) had a high risk for developing colorectal cancer compared with rural respondents (42.6%). One possible explanation for this is that previous research has linked urban residence to increased risk of CRC and incidence has been said to be consistently higher among urban residents (Janout and Kollárová, 2001). It further reported that current residence in an urban area is a stronger predictor of risk than is an urban location of birth (Janout and Kollárová, 2001). The finding from this current study is in contrast with a similar study in sub-urban Nigeria where 72% of participants had a high risk of developing colorectal cancer (Adeoti et al., 2016).

The findings of this work on risk factors of colorectal cancer was consistent with previous studies (Adeoti et al., 2016; Sessa et al., 2008) it showed that only 52 (8.57%) of rural and urban respondents had a family history of cancer, which is in accordance with a study in Saudi Arabia that reported family history of cancer among 8% of the respondents and Nigeria where 9.8% reported a family history of cancer (Adeoti et al., 2016; Osama et al., 2015). Urban respondents (9.9%) frequently consume alcohol in comparison to rural respondents (2.6%) and this agrees with the study among sub-urban respondents in Nigeria, which reported that 10% respondents in Osun state take alcoholic drink (Adeoti et al., 2016). One-third (31.91%) of urban respondents eat a diet low in fiber always and is in accordance with a study carried out in Saudi-Arabia among urban respondents that reported 28% of respondents eat diet low in fiber (Osama et al., 2015). While less than 5% of rural respondents eat diet low in fibre. This finding reflects the attributes associated to urban life that is indicated in their poor food choices and availability of unhealthy food, as explained by (Irabor, 2014) in the study carried out in Nigeria, economic development is an important environmental influence and is related to changes in dietary preferences that result from increased affluence. This reflects in the findings from the current study where more of urban respondents (40%) eat diet high in fat daily compared to 15.18% of rural respondents and one-fourth of urban respondents 73 (24.01%) are overweight compared with one-sixth of rural respondents 49 (16%). Ordinarily, we know that the native Nigerian diet consists of a bolus-type high fiber meal, with a vegetable-based stew assisting its swallowing. With globalization and adoption of Western diets, fewer native Nigerians still partake of their native diets as revealed among the urban residents in the study (Irabor, 2012; Irabor, 2014).Ultimately this finding will help in disseminating information on risk factors of CRC.

Preventive practice is birthed by knowledge (Myers et al., 2007) as revealed by the low level of prevention reported in the current study in which about one-fifth percent of rural respondent 21 (6.9%) take preventive actions against colorectal cancer in comparison to a slightly higher percent 55 (10.1%) of urban respondents who do something to prevent CRC, this represents less than 20% of respondents in the rural and urban local government. This simply shows that majority of respondents in rural and urban area don’t take preventive actions against colorectal cancer and this proves the fact that prevention measure does not completely has to do with rural or urban residence, but a major determinant is knowledge which showed in the low level of knowledge recorded in this study. For the rural area, just 2 (0.07%) respondents had previous history of CRC screening compared to 8 (2.6%) respondents in the urban area, this is in accordance with a study carried out in East Iran among urban respondents (Bidouei et al., 2014) that recorded only 4.2% of respondents reported prior screening for CRC and other 95.8% had never been tested before, in a similar study carried out in Nigeria in a sub-urban area, 9% reported previous CRC screening (Adeoti et al., 2016). Similarly, a study carried out in USA among rural and urban respondents also reported screening was significantly less likely among males, African–Americans, respondents living in rural-designated areas, those with younger age, those who had not graduated from high school but reported 74% of urban respondents ever being screened for CRC with the majority (70%) indicating colonoscopy (Heather et al., 2012; Bennett et al., 2012), which contradicts with the report on urban residents from the current study.

A significantly higher number of urban respondents (8.2%) prevent CRC by maintaining healthy weight in comparison to 4% of rural respondents (p=0.03). The low number of respondent is in agreement with a study carried out in urban setting in Saudi-Arabia where 28% modified their diet to prevent CRC and 20% modified their physical activity to guard against colorectal cancer (Osama et al., 2015) while in a study in Malaysia among rural respondent’s low prevention was recorded (Tin and Jun, 2013).

It could be suggested based on the result that respondents require more information about the disease which will result in better practice of preventive measures. Many developed nations have good public awareness campaigns that are specifically designed to improve education on CRC. This type of initiative is lacking in many developing countries due to poor support from funding bodies or participations from disease survivors.Mass media influences may also not be directed towards this course.

Demographic factors associated with the knowledge level of rural and urban respondents showed that knowledge of CRC was not significantly associated with age in either of the LGA’s. This is in contrast with findings from a similar study on knowledge and perception in Jordan and Asia that reported that better knowledge score was attributed to older age (Koo et al., 2012; Hana et al., 2015). In the urban area gender significantly associated with knowledge of CRC (p=0.014) although not for rural respondents, female and male were both knowledgeable (50%) each for the rural residents while male urban respondents (57.39%) were more knowledgeable than female (42.61%). This contradicts with a previous study that reported that there was no significant association with participant gender (Koo et al., 2012; Hana et al., 2015).Education was significantly associated with knowledge of CRC for rural residents (p=0.010) and urban residents (p<0.001) respectively, knowledge increased with education for both groups, this is in line with a study carried out in Saudi-Arabia among mixed respondents that reported correct answers are influenced by variables which includes higher educational levels. Furthermore, findings have consistently associated higher education with good CRC knowledge grade as revealed by the study (Yasmine et al., 2016; Osama et al.,2015; Taha et al., 2015; Vui et al., 2015). Occupation was significantly associated with knowledge level of CRC (p=0.001) for urban respondents, however not significantly associated for rural respondents (p=0.232), majority of urban respondents (68.7%) engaged in skilled occupation had good grades compared to rural respondents (51.5%). This is in contradiction to a study carried out in Hong-kong that reported poorer knowledge among employed respondents with the explanation they were more occupied with their own job duties and could be less aware of educational initiatives on CRC, and hence less knowledgeable (Coughlin and Thompson, 2004).

In conclusion, this study has revealed that the level of knowledge of sign/symptoms, risk factors and preventive measures of colorectal cancer was generally poor among the study groups, but significantly lower among rural respondents. Presence of risk- factor was higher among urban respondents while preventive practice was poor in both groups.

This is one of the few studies that compared knowledge, risk-factors and preventive practices for colorectal cancer among rural and urban respondents in Nigeria, However, it does have some limitations. Firstly, only one rural and one urban LGA were selected in the State so the findings may not be wholly representative of the entire population of the State, Also, data was dependent on respondents’ recall ability and no objective measures were used to verify most of the information. Third, we did not screen respondents for early signs of CRC and as such were unable to make comparisons of CRC in both groups. Further research may include actual screening of respondents in rural and urban areas. The underlying reasons for the poor levels of knowledge and preventive practices may also need to be explored.

## Funding

No external funding.

## Statement conflict of Interest

None.

## References

[B1] Abdulkareem FB, AbuduEk, Awolola NA (2008). Colorectal carcinoma in Lagos and Sagamu, Southwest Nigeria: A histopathological review. World J Gastroenterol.

[B2] Adeoti ML, Oguntola SA, Olugbenga-Bello AI, Oladimeji OJ, JegedeSO (2016). Colorectal cancer: Knowledge and risk factors among adults in a Suburban Nigerian Community. JMSCR.

[B3] Akunnaya PO, Adedapo O (2014). Trends in urbanisation: Implication for planning and low-income housing delivery in Lagos, Nigeria. Archit Res.

[B4] Aria Jefferson Health (2017). Home of Sidney kimmel medical college.

[B5] Bennett KJ, Probst JC, Bellinger JD (2012). Receipt of cancer screening services: surprising results for some rural minorities. J Rural Health.

[B6] Berkowitz Z, Hawkins NA, Peipins LA (2008). Beliefs, risk perceptions, and gaps in knowledge as barriers to colorectal cancer screening in older adults. J Am Geriatr Soc.

[B7] Bidouei F, Abdolhosseini S, Jafarzadeh N (2014). Knowledge and perception toward colorectal cancer screening in east of Iran. Int J Health Policy Manag.

[B8] Celik AP, Zyman R, Mahdi R (2009). Sustainable urbanization in the information age ST/ESA/ PAD/SER.E/137, Department of Economic and Social Affairs Division for Public Administration and Development Management.

[B9] Center of disease and control CDC (2008). Colorectal Cancer Statistics. 2008.

[B10] Chalya PL, McHembe MD, MabulaJB (2013). Clinicopathological patterns and challenges of management of colorectal cancer in a resource-limited setting, a Tanzanian experience. World J Surg Oncol.

[B11] Christou A, Thompson S (2012). Colorectal cancer screening knowledge, attitudes and behavioural intention among Indigenous Western Australians. BMC Public Health.

[B12] Clinical, histological and immunohistochemical (2014). Study Of Colorectal Carcinoma Colorectal cancer facts &amp; figures 2014-2016. Atlanta: American Cancer Society, 2014.

[B13] Correa P, Haenszel W (1987). The epidemiology of large-bowel cancer. Adv Cancer Res.

[B14] Coughlin SS, Thompson TD (2004). Colorectal cancer screening practices among men and women in rural and nonrural areas of the United States, 1999. J Rural Health.

[B15] Hana T, Madi A, Musa S, Sireen A, Vanja B (2015). Knowledge and perceptions about colorectal cancer in Jordan. Asian Pac J Cancer Prev.

[B16] Heather MB, Heather RD, Patricia AS (2012). Relationship of colorectal cancer awareness and knowledge with colorectal cancer screening. Colorectal Cancer.

[B17] Irabor DO (2014). Diet, environmental factors and increasing incidence of colorectal cancer in Nigeria. Ann Nigerian Med.

[B18] Irabor DO (2012). Ethnic differences in colon and rectal cancer incidence in Nigeria: A case of dietary determinants?. Ann Nigerian Med.

[B19] Janout V, Kollárová H (2001). Epidemiology of colorectal cancer. Biomed Pap Med Fac Univ Palacku Olomouc Czech Repub.

[B20] Joseph DA, King JB, Miller JW (2012). Prevalence of colorectal cancer screening among adults-behavioral risk factor surveillance system, United States, 2010. Morb Mortal Wkly Rep.

[B21] KarimAlwan A (2017). Principles and practice of cancer prevention and control.Faculty of Medicine, Segi University, Malaysia.OMICS ebook group.

[B22] Koo JH, Leong RW, Ching J (2012). Knowledge of, attitudes toward, and barriers to participation of colorectal cancer screening tests in the Asia-Pacific region: a multicenter study. Gastrointest Endosc.

[B23] McCaffery K, Wardle J, Waller J (2003). Knowledge, attitudes, and behavioral intentions in relation to the early detection of colorectal cancer in the United Kingdom. Prev Med.

[B24] Muggah R (2012). Researching the Urban Dilemma: Urbanization, Poverty and Violence.

[B25] Myers RE, Sifri R, Hyslop T (2007). A randomized controlled trial of the impact of targeted and tailored interventions on colorectal cancer screening. Cancer.

[B26] Ng S, Wong S (2013). Colorectal cancer screening in Asia. Br Med Bull.

[B27] Obayelu AE, Okoruwa VO, Oni OA (2009). Analysis of rural and urban households’ food consumption differential in the North-Central, Nigeria: A micro-econometric approach. J Dev Agric Econ.

[B28] Onibokun A, Faniran A (2015). Urbanization and Urban Problems in Nigeria. Urban Research in Nigeria.

[B29] Osama AW, Fahad A, Arwa Mohammed A, Kassim AK, Ahmed KI (2015). Colorectal cancer risk factors: A study of knowledge, attitude, and practice among adults in Riyadh, Saudi Arabia. Cancer Res J.

[B30] Sessa A, Abbate R, Di Giuseppe G, Marinelli P, Angelillo IF (2008). Knowledge, attitudes, and preventive practices about colorectal cancer among adults in an area of Southern Italy. BMC Cancer.

[B31] Sung JJ, Choi SY, Chan FK (2008). Obstacles to colorectal cancer screening in Chinese: a study based on health belief model. Am J Gastroenterol.

[B32] Tin T, Jun YG (2013). Level of colorectal awareness; a crossectional study among multi-ethnic rural population in Malaysia. Biomed Central. BMC Cancer.

[B33] Von Wagner C, Knight K, Steptoe A, Wardle J (2007). Functional health literacy and health-promoting behaviour in a national sample of British adults. J Epidemiol Commun Health.

[B34] Vui HC, Ai GL, Hana NB, Jackson T, Chee FC (2015). Poor knowledge of colorectal cancer in Brunei Darussalam. Asian Pac J Cancer Prev.

[B35] Wolf MS, Rademaker A, Bennett CL (2005). Development of a brief survey on colon cancer screening knowledge and attitudes among veterans. Prev Chronic Dis, [serial online] 2005 April 04. http://www.cdc.gov/pcd/issues/2005/apr/04_0104.htm.

[B36] World Health Organization (2012). Cancer fact sheet. [online].

[B37] Yasmine SG, TarekTawfik A, Abdulelah KA (2016). Colon cancer among older Saudis: Awareness of risk factors and early signs, and perceived barriers to screening. Asian Pac J Cancer Prev.

